# Pulse Increase of Soil N_2_O Emission in Response to N Addition in a Temperate Forest on Mt Changbai, Northeast China

**DOI:** 10.1371/journal.pone.0102765

**Published:** 2014-07-31

**Authors:** Edith Bai, Wei Li, Shanlong Li, Jianfei Sun, Bo Peng, Weiwei Dai, Ping Jiang, Shijie Han

**Affiliations:** 1 State Key Laboratory of Forest and Soil Ecology, Institute of Applied Ecology, Chinese Academy of Sciences, Shenyang, China; 2 College of Resources and Environment, University of Chinese Academy of Sciences, Beijing, China; Tennessee State University, United States of America

## Abstract

Nitrogen (N) deposition has increased significantly globally since the industrial revolution. Previous studies on the response of gaseous emissions to N deposition have shown controversial results, pointing to the system-specific effect of N addition. Here we conducted an N addition experiment in a temperate natural forest in northeastern China to test how potential changes in N deposition alter soil N_2_O emission and its sources from nitrification and denitrification. Soil N_2_O emission was measured using closed chamber method and a separate incubation experiment using acetylene inhibition method was carried out to determine denitrification fluxes and the contribution of nitrification and denitrification to N_2_O emissions between Jul. and Oct. 2012. An NH_4_NO_3_ addition of 50 kg N/ha/yr significantly increased N_2_O and N_2_ emissions, but their “pulse emission” induced by N addition only lasted for two weeks. Mean nitrification-derived N_2_O to denitrification-derived N_2_O ratio was 0.56 in control plots, indicating higher contribution of denitrification to N_2_O emissions in the study area, and this ratio was not influenced by N addition. The N_2_O to (N_2_+N_2_O) ratio was 0.41–0.55 in control plots and was reduced by N addition at one sampling time point. Based on this short term experiment, we propose that N_2_O and denitrification rate might increase with increasing N deposition at least by the same fold in the future, which would deteriorate global warming problems.

## Introduction

Anthropogenic nitrogen (N) has approximately doubled the input of reactive N to the Earth's land surface since the industrial revolution [1], [2]. There are three potential fates of anthropogenic N entering terrestrial ecosystem: loss via nitrification and denitrification to the atmosphere as gases, loss via leaching into the aquatic ecosystems, and storage in plants and soils [3]. The gaseous products include NO_x_, N_2_O, N_2_ and NH_3_, among which N_2_O is a potent greenhouse gas [4] and ozone-depleting agent [5] and has been a major concern for environmental scientists. Although previous studies suggested that many ecosystems have high N retention capacity without evidence of adverse effects of increasing N deposition [6], some other studies found a big increase of N_2_O following increase of N inputs [7]–[9]. Therefore, the response of gaseous emissions to N deposition may be system-specific and depends on the N status [10] and environmental factors of the ecosystem [11].

Soil N_2_O flux has been found to be affected by ammonium and nitrate availability [12], water filled pore space (WFPS) [13], soil temperature [14], soil moisture[15], soil pH [16], and carbon (C) availability [17]. When increasing N deposition increases inorganic N availability for nitrification and denitrification, it is expected to increase N_2_O emission [18]. However, inorganic N availability may not increase in response to increasing N deposition due to plant uptake and microbial immobilization [6]. In addition, soil pH has been found to decrease in response to increasing N deposition, which may lower N_2_O emissions [19]. Decomposition of light fractions of soil C with low density (<1.7 g cm^−3^) has been found to accelerate under long term N addition [20], which may affect C availability for nitrification and denitrification and thereby N_2_O emission.

N_2_O is derived from both nitrification and denitrification and it is important to distinguish these processes in order to better understand factors controlling the emission of N_2_O [21]. The contribution of the two processes is mainly affected by oxygen availability and forms and availability of inorganic N [22], [23]. Therefore, increasing N deposition may impact the ratio of nitrification derived to denitrification derived N_2_O. However, very limited data exist on this ratio or the denitrification rate in temperate forest ecosystems [3], [24], [25].

This study was carried out in a broad-leaved Korean pine (*Pinus koraiensis* Siebold & Zucc) mixed forest, which is the dominant natural forest type in northeastern China. The system was previously thought to be N-limited but may be experiencing N saturation currently [26]. Hence, it is important to assess how increasing N deposition may affect the N cycling of this forest type. The objectives of our study were: 1) to measure soil N_2_O fluxes, ratio of nitrification derived to denitrification derived N_2_O, and denitrification rate, for the first time in the study area; 2) to examine the treatment effect of N addition on these fluxes; 3) to investigate the influences of environmental factors on these variables. The hypotheses of this study are: 1) N addition would increase soil N_2_O emissions and denitrification rate; 2) soil temperature, moisture, WFPS, pH, and soil texture play an important role in soil N_2_O emissions and denitrification.

## Materials and Methods

The study was carried out within the research forest of Changbai Forest Ecosystem Research Station (CBFERS) established in 1979 (127°38' E, 41°42' N). No specific permits were required for the described field studies. Research activities within that research forest do not need any specific permissions from any government levels, but need to inform Professor Han (Director CBFERS, co-author of the present paper). The field studies did not involve endangered or protected species.

### Study area

The study region is characterized by a typical temperate climate, with long and cold winters, and warm summers. Mean annual temperature is 3.6 °C, with the highest temperature in July, and the lowest in January. Mean annual precipitation is 745 mm, mainly falling between May and September. The growing season is from Jun. to Sep., with mean temperature at 16.7 °C. Natural Korean pine and broad-leaved mixed forest is distributed from approximately 750 to 1100 m asl. The soil is a dark brown soil developed from volcanic ash (Albic Luvisol).

### Experimental design

Present N deposition rate in China is 12.89–63.53 kg N ha^−1^ yr^−1^, with higher values in eastern China [27]. It is predicted that in 2050, many areas in eastern China will have a deposition rate of 50 kg N ha^−1^ yr^−1^ [1], [28]. Current N deposition rate in the study area is 23 kg N ha^−1^ yr^−1^ [26], and we chose a N addition rate of 50 kg N ha^−1^ yr^−1^ in the treatment as a reasonable estimate of future change. Six sampling plots (25 m×50 m) were established: three replicates for both control and N addition treatment. Plots were separated by 10 m buffer strips. Vegetation was homogeneous based on visual observation.

Starting in 2008, NH_4_NO_3_ solution has been evenly sprayed for N addition treatment monthly from May to September at a rate of 10 kg N ha^−1^ (starting on May 17^th^), giving an annual N addition rate of 50 kg N ha^−1^ yr^−1^. The same volume (40 L) of water was sprayed on the control plots.

### Gas sampling and analysis

Soil N_2_O emission was measured using closed chamber method [29]. A steel-made chamber (0.5 m×0.5 m×0.5 m) with a septum for gas sampling was placed on the base every time gases were sampled. The bases were installed into the soil one month before initial gas sampling to avoid disturbance on soils. Water was used to seal the connection between the chamber and the base. The chamber was covered with thermal insulation cotton to avoid temperature increase in the chamber. Fans were equipped in the chamber to increase air circulation. Gas sample was collected from each chamber every 30 mins for 2 hours and stored in air bags for measurement.

We sampled for three N addition periods in year 2012, 5–6 times for each period. Samples were taken once every 5–6 days. Given that NH_4_NO_3_ was sprayed on the day 17^th^ each month, gas samples were collected in three periods: Period one (Jul. 17^th^–Aug. 16^th^): 7, 9, 13, 18, and 27 days after N addition; Period two (Aug. 17^th^–Sep. 16^th^): 4, 8, 15, 19, 24, and 28 days after N addition; Period three (Sep. 17^th^ to Oct. 18^th^): 3, 9, 17, 22, 26, and 31 days after N addition. Samples were taken between 9:00 and 11:00 hours and between 15:00 and 17:00 hours because during these two time slots the flux can represent daily average [30]. N_2_O concentration was measured by gas chromatography (HP 5890-II). The temperature of the chromatographic column was 55°C and the carrying gas was high purity N_2_.

Acetylene inhibition method [31], [32] was used to determine denitrification rates and to differentiate sources of N_2_O based on the following principle: high concentration of acetylene (10 kPa) could inhibit both nitrification and the reduction of N_2_O to N_2_ by denitrifiers, so that the otherwise emitted N_2_ remained as N_2_O. When the acetylene concentration was low (10–100Pa), only nitrification was inhibited [33]. Emitted N_2_O could be from three sources: N_2_O_den_ (N_2_O produced by denitrification); N_2_O_N2_(additional N_2_O produced by denitrification, instead of normal N_2_ production); N_2_O_nit_ (N_2_O produced by nitrification). In each sampling plot, soils were incubated *in situ* in three tightly sealed bottles injected with different concentrations of C_2_H_2_ (purified by H_2_SO_4_ and distilled water): 0 (nothing inhibited), 60 Pa (nitrification inhibited), and 10 kPa (nitrification and the reduction of N_2_O to N_2_ inhibited). Correspondingly, measured N_2_O of the three incubation bottles were N_2_O_nit_+N_2_O_den_, N_2_O_den_, and N_2_O_den_+N_2_O_N2_, respectively. Therefore, nitrification-derived N_2_O, denitrification-derived N_2_O, and denitrification-derived N_2_ can be calculated from these three measured N_2_O fluxes. Denitrification rate is equal to N_2_O_den_+N_2_O_N2_.

60 Pa C_2_H_2_ was used for low concentration treatment as recommended in previous experiment in the study area [34]. This concentration has also been used in other experiments [35], although 10 Pa is more widely used.

Although the acetylene inhibition technique has not always been satisfactory due to incomplete or non-specific inhibition [36], at present it is still a widely-used method for obtaining a reasonable estimate of denitrification rate and ratio of nitrification to denitrification derived N_2_O [3], [37]–[40]. We used an *in situ* incubation method to reduce some potential bias [41].

Three sampling points were selected within each sampling plot. Nine soil cores (5 cm diameter×10 cm depth) were sampled by PVC tubes at each point. There were 25 holes (0.6 cm diameter) on each PVC tube to ensure acetylene circulation. At each sampling point, every three soil cores were assigned to one of the three incubation bottles. Therefore, we had three soil cores in each incubation bottle at each plot. Before the incubation, the headspace air was replaced by purified C_2_H_2_. The bottles were then buried *in situ* and 60 ml headspace samples were taken after 24 hours incubation and analyzed for N_2_O concentration by gas chromatography (HP 5890-II). Gas samples were collected on Aug. 18^th^, Aug. 30^th^, Sep. 10^th^, Sep. 16^th^, Sep. 23^rd^, Sep. 30^th^, Oct. 7^th^, and Oct. 15^th^, 2012.

### Soil sampling and analysis

**Figure 1:**
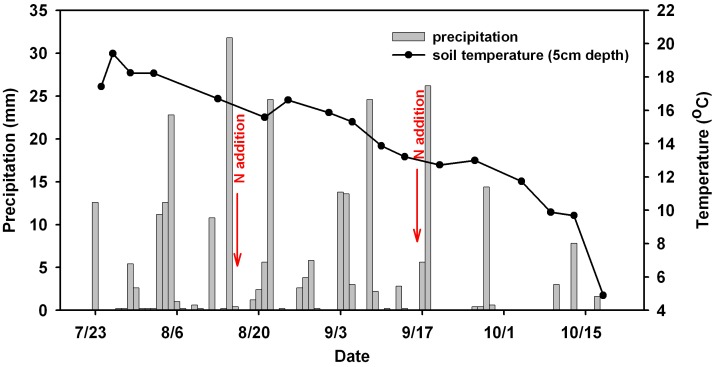
Variations of precipitation and soil temperature (5 cm depth) in the study area during the sampling periods.

Air temperature and soil temperature at 5 cm depth were recorded *in situ* at each sampling time of both closed chamber experiment and acetylene inhibition experiment using TP3001 thermometer (Boyang, China) (Fig. 1). Precipitation was recorded at the local weather station in the Changbai Mountain Nature Reserve (Fig. 1). Bulk density (BD) in each sampling plot (n = 3) was determined by the core method [42].

One soil sample (0–10 cm) in each plot was collected at each sampling time to determine pH, soil moisture content, and soil NH_4_^+^ and NO_3_^-^. Standard oven-drying method was used to measure soil gravimetric water content, which was expressed as kg H_2_O/kg dry soil. WFPS was calculated based on Eqn.1:
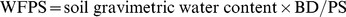
(Eqn.1)where PS is soil pore space and is calculated from Eqn.2 [43]:
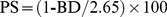
(Eqn.2)20 g fresh soil was passed through 2 mm screen and extracted by 50 mL 2 M KCl. The solution was shaken for 1 hour and filtered through rapid filtering paper. Soil inorganic N in extracts was determined by Futura continuous flow analysis system (Alliance, France).

Soil pH was measured on a 1∶2.5 soil solution in de-ionized water using a pH meter. Soil texture was determined by the pipette-sedimentation method [44].

### Statistical analysis

Repeated measures ANOVA was used to examine the effects of N addition using N treatment method as the between subject factor and sampling time as the within subject factor. All analyses were carried out in SPSS [45].

## Results

### N_2_O fluxes

Soil characteristics of the sampling plots were tested 17 times from Jul. to Oct. 2012 and mean results were reported in Table 1 (values were reported as mean ± standard deviation). Mean soil pH was 5.48±0.33 for control plot and 5.47±0.09 for N-treated plot. Soil clay content was 31.67±4.40% and 30.98±1.11% for control and N-treated plot, respectively. Soil bulk density was also similar between control and N-treated plots (0.581±0.013 g cm^−3^ and 0.587±0.113 g cm^−3^ respectively). Soil moisture and WFPS were not significantly different between N-treated plots (101.90±26.94 kg H_2_O kg^−1^ dry soil and 76.07±20.15% respectively) and control plots (94.81±21.99 kg H_2_O kg^−1^ dry soil and 70.48±15.88% respectively). Mean soil NO_3_^-^ concentration was significantly increased in N-treated plots (22.56±26.26 mg N kg^−1^ soil) compared to control plots (19.11±23.87 mg N kg^−1^ soil) based on repeated measures ANOVA, but mean soil NH_4_^+^ concentration was not changed (Table 1).

**Table 1:** Soil characteristics of treatment plots.

	pH	Clay (%)	Bulk Density (g/cm^3^)	WFPS (%)	Soil T (°C)	Soil moisture (kg H_2_O/kg dry soil)	NH_4_^+^ (mg N/kg soil)	NO_3_^-^ (mg N/kg soil)	Inorganic N (mg N/kg soil)
Control	5.48±0.33^a^	31.67±4.40^a^	0.581±0.013^a^	70.48±15.88^a^	14.25±3.67^a^	94.81±21.99^a^	12.50±9.79^a^	19.11±23.87^a^	31.62±22.39^a^
N-treated	5.47±0.09^a^	30.98±1.11^a^	0.587±0.113^a^	76.07±20.15^a^	14.25±3.67^a^	101.90±26.94^a^	10.40±8.93^a^	22.56±26.26^b^	32.96±22.47^b^

Soils were collected 17 times from Jul. to Oct. 2012. Values are reported as mean ± standard deviation. WFPS: water-filled pore space. T: temperature. Sampling time was used as the within subject factor and N treatment was used as the between subject factor in repeated measures ANOVA. Different letter represents significant difference (p = 0.05) caused N treatment.

**Figure 2:**
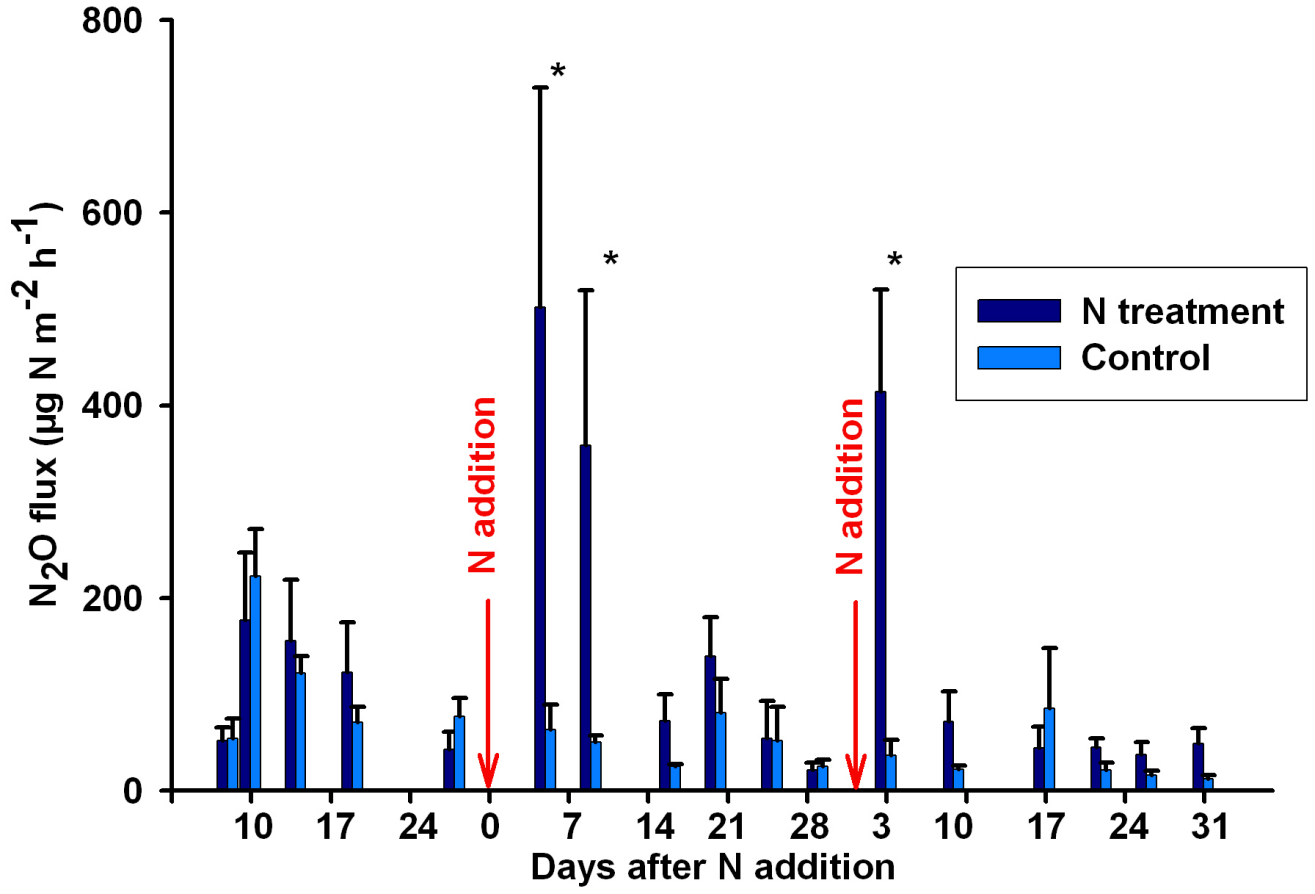
Effects of N addition on N_2_O fluxes (n = 3). Arrows represent N addition on that date. Error bars are standard errors. * represents significant effect at p = 0.05.

N-treated plots had significantly higher N_2_O fluxes right after N addition for period 2 and 3 (Fig. 2). For example, four days after N addition in period 2, mean N_2_O emission in N-treated plots reached 501.74 µg N m^−2^ h^−1^, which was significantly higher than that in control plots (63.31 µg N m^−2^ h^−1^). This difference between N-treated and control plots declined with time and became minimal on the 15^th^ day after N addition. Similarly, for the next sampling period, mean N_2_O emission in N-treated plots was 414.07 µg N m^−2^ h^−1^ three days after N addition, compared with 36.88 µg N m^−2^ h^−1^ in control plots. Only 17 days later, N_2_O flux was similar in N-treated (44.13 µg N m^−2^ h^−1^) and control (85.38 µg N m^−2^ h^−1^) plots.

Pearson's correlation analysis showed that N_2_O emission was positively correlated with soil NO_3_^-^ (r = 0.225), soil total inorganic N (r = 0.210), and soil temperature (r = 0.253) (Table 2). N_2_O emission first increased with WFPS then decreased, and the highest N_2_O flux rate was observed at 80% WFPS. Soil pH was positively correlated with soil clay content (r = 0.727) (Table 2).

**Table 2:** Pearson's correlation coefficients between N_2_O fluxes and soil characteristics.

Parameter	N_2_O µg N/m^2^/h	Soil M (kg H_2_O/kg dry soil)	WFPS (%)	NH_4_^+^ (mg N/kg soil)	NO_3_^-^ (mg N/kg soil)	Inorganic N (mg N/kg soil)	pH	Clay (%)	Soil T (°C)
N_2_O	1	−0.108	−0.028	−0.103	0.225*	0.210*	0.050	−0.072	0.253*
Soil M		1	0.797*	−0.023	0.079	0.079	0.099	0.169	−0.113
WFPS			1	−0.021	0.144	0.153	0.085	0.023	−0.109
NH_4_^+^				1	−0.475*	−0.116	−0.015	−0.027	0.016
NO_3_^−^					1	0.929*	−0.042	−0.014	0.460*
Inorganic N						1	−0.054	−0.027	0.526*
pH							1	0.727*	0.000
Clay								1	0.000

WFPS: water-filled pore space. T: temperature. M: moisture. * represents significant correlation at p = 0.05.

### Sources of N_2_O and denitrification flux

**Figure 3:**
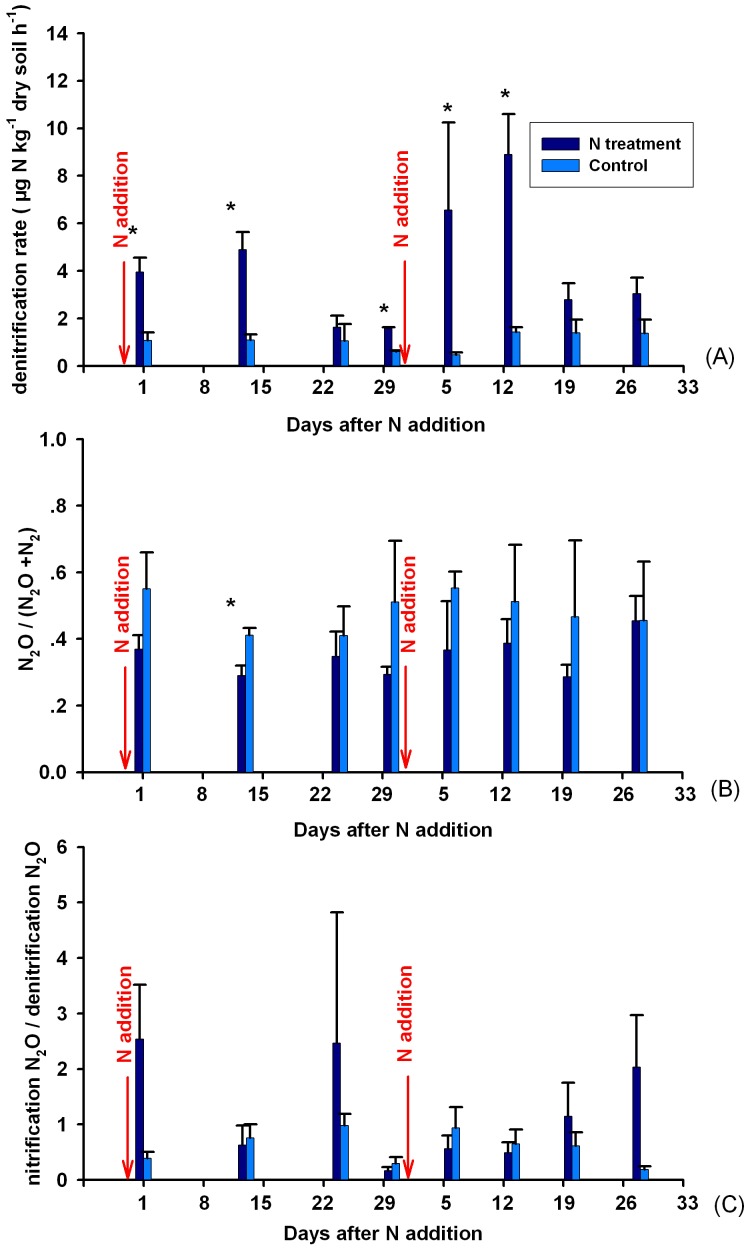
Effect of N addition on denitrification rate (A), N_2_O: (N_2_O+N_2_) ratio (B), and nitrification-derived N_2_O: denitrification-derived N_2_O ratio (N_2_O_nit_:N_2_O_den_) (C) (n = 3). Arrows represent N addition on that date. * represents significant effect at p = 0.05.

One day after N addition in August, denitrification rate was significantly higher in N-treated plots (3.95 µg N kg soil^−1^ h^−1^) than in control plots (0.37 µg N kg soil^−1^ h^−1^) (Fig. 3a). The effect lasted till the end of the treatment cycle. The same phenomenon was observed for the next sampling period, except that the effect of N addition was not statistically significant on the last two sampling dates (Fig. 3a). The N_2_O to (N_2_+N_2_O) ratios in N-treated plots (0.29–0.45) were all lower than those in control plots (0.41–0.55), although the effect was not statistically significant (Fig. 3b). N-additions on Aug. 17^th^ and Sep. 17^th^ did not affect this pattern (Fig. 3b). We did not find a general pattern for the temporal variation of the N_2_O_nit_: N_2_O_den_ ratio or a significant effect of N addition on the ratio (Fig. 3c). The N_2_O_nit_: N_2_O_den_ ratio in N-treated plots had a wider range (0.17–4.47) than that in control plots (0.19–0.94).

Correlation analysis indicated both denitrification rate and N_2_ flux had a significantly positive correlation with WFPS (r = 0.344 and 0.353 respectively). Neither the N_2_O to (N_2_+N_2_O) ratio nor the N_2_O_nit_: N_2_O_den_ ratio correlated with WFPS or any other measured soil variables (Table 3).

**Table 3:** Pearson's correlation coefficients between soil characteristics and denitrification rate, N_2_ emission rate, N_2_O/(N_2_O+N_2_), and nitrification-derived N_2_O: denitrification-derived N_2_O (N_2_O_nit_:N_2_O_den_) ratios.

	Denitrification µg N/kg soil/h	N_2_ µg N/kg soil/h	N_2_O/(N_2_O+N_2_)	N_2_O_nit_:N_2_O_den_	WFPS (%)	NO_3_^-^ (mg N/kg soil)	Inorganic N (mg N/kg soil)	pH	Clay (%)	Soil T (°C)	Soil M (kg H_2_O/kg dry soil)
Denitrification	1	0.965^*^	−0.306^*^	−0.035	0.344^*^	0.202	−0.001	0.018	−0.092	0.030	0.273
N_2_		1	−0.379^*^	−0.013	0.353^*^	0.191	0.006	0.054	−0.098	0.049	0.144
N_2_O/(N_2_O+N_2_)			1	−0.173	−0.054	−0.131	−0.088	−0.107	−0.050	−0.045	−0.122
N_2_O_nit_:N_2_O_den_				1	−0.054	−0.061	0.045	0.008	0.094	0.029	0.196
WFPS					1	0.249	−0.028	0.131	0.013	−0.237	0.672^*^
NO_3_^-^						1	0.659^*^	0.019	−0.115	0.136	0.137
Inorganic N							1	0.072	−0.017	0.523^*^	0.022
pH								1	0.727^*^	0.000	0.105
Clay									1	0.000	0.177
Soil T										1	−0.225

WFPS: water-filled pore space. T: temperature. M: moisture. * represents significant correlation at p = 0.05.

## Discussion

### N_2_O emission and its sources

N_2_O emission fluxes observed in the control plots were between 0.77 and 382.75 µg N m^−2^ h^−1^, which mostly is within the range of soil N_2_O emission in a 40-year-old pine forest ecosystems in the USA (3–424 µg N m^−2^ h^−1^) [46]. Our results suggested N addition (50 kg N ha^−1^ yr^−1^) could increase N_2_O fluxes to up to 501 µg N m^−2^ h^−1^ and the monthly sum of N_2_O emission in N-treated plots were five-times higher than that in control plots (Fig. 2). The positive correlation between N input rate and the magnitude of N_2_O emissions has been found in previous studies [9], [11], [47]. Previously, Bowden et al. [48] reported N addition at 50–150 kg N/ha/yr only increased N_2_O emission from 0.2–1.1 to 0.7–5.2 µg N m^−2^ h^−1^ in the pine forest plantation of Harvard Forest, US, which is much lower than our results. However, our results are consistent with those observations that showed a big increase of N_2_O following increase of N inputs [7]–[9]. Our speculation on the discrepancy is that Harvard Forest in 1990s was N-limited and thereby could retain added N efficiently, while our forest in Changbai Mountain is experiencing “kinetic N saturation” currently [49], with lower N sinks than the N input rate.

N addition effect on N_2_O emissions diminished quickly with time after N addition, because added NH_4_^+^ and NO_3_^-^ were also subject to plant uptake, microbial immobilization, and leaching (Fig. 2). We found the increase of N_2_O emission after N addition only lasted for two weeks. Scheer et al. [7] also observed similar “N_2_O emission pulses” which lasted for 7 days after N-fertilizer application in combination with irrigation events in a cotton field. This temporal variation suggests more frequent sampling is necessary to capture the dynamics of the emission pulse following N addition.

Precipitation and soil texture, which determines WFPS, may play an important role in soil N_2_O emission. Positive correlations between WFPS and N_2_O were usually observed because the contribution of denitrification-derived N_2_O increases with WFPS [50], [51]. Other potential reasons include: microbes were water-limited [52]; rain may disrupt physical aggregate and increase soil organic matter exposure for mineralization and denitrification [53]; and water may alleviate diffusional constraints [54], [55]. However, when WFPS is very high (>80%), those factors were not limiting and N_2_O fluxes may decline due to their further reduction to N_2_ [56], [57]. Because our mean WFPS mainly varied between 55% and 100% (Table 1), N_2_O emissions increased with WFPS first and then decreased, and the highest N_2_O flux rate was observed at WFPS = 80%. We speculate this was because N_2_O from both nitrification and denitrification was high at this water and oxygen level.

We found N_2_O fluxes were positively correlated with soil temperature (Table 3), which is consistent with many previous findings [58]–[60]. Both nitrification-derived N_2_O [61] and denitrification-derived N_2_O [62], [63] have been found to be higher at lower pH in acid forest soils. However, nitrification-derived N_2_O has more often been found to increase with pH [19]. Our N addition treatment did not affect soil pH (Table 1) and N_2_O fluxes were not related to soil pH in our study (Table 2).

The N_2_O_nit_: N_2_O_den_ ratio was not affected by N addition (Table 3). The N_2_O_nit_: N_2_O_den_ ratio in N-treated plots had a larger variation (0.17–4.47) than that in control plots (0.19–0.94). Mean N_2_O_nit_: N_2_O_den_ ratio was 0.56 and 1.46 in control and N-treated plots respectively, which is lower than what Papen & Butterback-Bahl [47] found (2.33) in spruce and beech forest ecosystem in Germany. The WFPS in our area was high (Table 1), therefore more N_2_O was emitted from denitrification. The N_2_O_nit_: N_2_O_den_ ratio was not significantly correlated with any soil factors we examined (Table 3), which suggested that the effects of soil moisture, temperature, WFPS, and texture on N_2_O_nit_: N_2_O_den_ ratio may be non-linear, or negligible.

### Denitrification fluxes and the N_2_O to (N_2_+N_2_O) ratio

Denitrification in the control plots were between 0.126 and 5.460 µg N kg^−1^ soil h^−1^ with a mean at 1.581 µg N kg^−1^ soil h^−1^ (Fig. 3). Bulk density of the study site was between 0.53 and 0.67 g cm^−3^ (Table 1) with a mean at 0.60 g cm^−3^. Assuming denitrification keeps at the high flux rate during the growing season (May-September) and there is no denitrification in the other months [64], denitrification in the region was roughly estimated to be 0.346 g N m^−2^ yr^−1^. This is close to the upper end of the modelled average value (0.156–0.368 g N m^−2^ yr^−1^) in global temperate and boreal forest ecosystems by Bai et al. [65]. *In situ* denitrification data from temperate forest are very limited. Dannenmann et al. [66] reported a denitrification rate of 0.185–0.622 g N m^−2^ yr^−1^ in a mountainous beech forest using seasonally weighted means, although their direct up-scaling of plot means got much higher values (1.4–9.37 g N m^−2^ yr^−1^). Wolf & Brumme [62] got 0.088–0.351 g N m^−2^ yr^−1^ *in situ* denitrification rates from beech forest floor and mineral soils. Our estimation is comparable to these above reported values.

N addition significantly increased denitrification rates to a mean of 5.539 µg N kg^−1^ soil h^−1^ (Table 3, Fig. 3a). With the current N deposition rate at 23 kg N ha^−1^ yr^−1^ [26], an N addition of 50 kg N ha^−1^ yr^−1^ (2.2 fold) has caused approximately a 2.5 fold increase in denitrification. Therefore, we predict that with the increase of N deposition, denitrification will increase correspondingly, at least by the same fold.

For the two gaseous products of denitrification, more N_2_ is emitted after N addition compared with N_2_O because the N_2_O to (N_2_+N_2_O) ratio was lower in N-treated plots (Fig. 3b). The N_2_O to (N_2_+N_2_O) ratios were between 0.41–0.55 and 0.29–0.45 in control and N-treated plots respectively. The N_2_O to (N_2_+N_2_O) ratio has been used to estimate denitrification rate because N_2_O was easier to measure [3], [67]. Our data in control plots were close to the mean value in natural soils (0.43–0.56) compiled by Schlesinger [3]. Increased NO_3_^-^ has been found to increase the N_2_O to (N_2_+N_2_O) ratio because of the greater affinity of NO_3_^-^ as terminal electron acceptor [68]–[70]. It is not clear why N addition slightly reduced the N_2_O to (N_2_+N_2_O) ratio in our study, and further exploration is needed.

WFPS was positively correlated with N_2_ emission, but not with N_2_O fluxes (Table 2, Table 3). This is because the relationship between WFPS and N_2_ fluxes is unidirectional, while bell-shaped relationship between WFPS and N_2_O is more common [13].

In conclusion, N addition did not change soil pH or soil bulk density in the study area. Mainly due to the increase of substrate availability, N addition significantly increased N_2_O and denitrification fluxes. However, the effect only lasted for two weeks. Based on this short term experiment, we propose that denitrification rate might increase at least by the same fold as the increase of N deposition in the future. N_2_O may also increase with increasing N deposition in our studied forest, deteriorating global warming problems. Future studies on the underlying mechanism [71] behind increasing N_2_O emissions following N addition are needed to better understand the processes contributing to increasing N_2_O emissions.
